# The Study of Medicine

**Published:** 1823-03-01

**Authors:** 


*The Study of Medicine.* By JOHN MASON GOOD, M.D. &c. &c. In Four Volumes, 8vo.

[Second Analytical Article, contined from page 596 ]

### CLASS II.—PNEUMATICA;

### OR DISEASES OF THE RESPIRATORY FUNCTION.

IN pursuance of our plan, we come now to another class of vital functions, hardly less important in itself than the first; but, embracing a very limited number of diseases in our author's system. This class is divided into two orders—the first termed Phonica, or affections of the vocal avenues—the second Pneumatica, or affections of the parenchymatous structure, membranes, and motive powers of the lungs. The anatomico-physiological proem to this class, occupies about 25 pages, and does not offer any thing on which we could make any useful remark. We question whether *ventriloquism* should have usurped an eighth of the whole proem—it does occupy three pages and a half out of twenty-five. We must also pass over the whole of the first order, embracing the six following genera:—viz. Coryza, Polypus, Rhonchus, Aphonia, Dysphonia, and Psellismus. These subjects occupy a space of about 50 pages, and afford a considerable stock of curious, and not uninteresting, information.

The second order is also divided into six genera, viz. Bex, Dyspnœa, Asthma, Ephialtes, Sternalgia, and Pleuralgia.

We observe that Dr. Good has considered cough (Bex) as, "at times, as truly idiopathic as any complaint whatever." We confess that we can hardly look upon it as any thing else than a mere symptom of a variety of affections. Indeed, in the greater number of instances, we consider it not a disease, but a remedy, or, at least, an effort of Nature to get rid of some offending matter. In all other cases, we look upon it as the effect of irritation propagated, directly or indirectly, on the air-passages—and, consequently, in every instance, *symptomatic*. Thus, in Dr. Good's first species, BEX HUMIDA, or common cough, "the exhalents (he says) of the bronchiæ are stimulated by an irritation of some kind or other, frequently by a reverse sympathy, &c. whence the bronchial vessels become overloaded and relieve themselves by an expectoration, &c." Surely the cough, or effect of this morbid state of the bronchial vessels should not be nominated the disease itself. The same reasoning will apply to all his other species of cough—even to the BEX CONVULSIVA, or hooping cough, in which "there can be little doubt that it proceeds, in most instances, from a miasm of a specific nature and peculiar character, having a direct determination to the lungs." Now it will be readily allowed, that the miasm is received into the body long before the cough begins—and, that some change must take place in the mucous membrane of the lungs before the cough is excited—in short, the disease itself must have actually commenced some time before the phenomenon of cough exists, and then, a single symptom is put down for the whole disease! This is one of the imperfections of nosology.

Dr. Good enters little, if at all, into the pathology of the disease, unless the following passage can be considered in that light.

> "In infants, it is mostly alarming from its tendency to produce convulsions, suffocation, apoplexy, inflammation of the brain, ruptures, and incurvation of the spine. In adults, it excites pneumonitis more frequently than in children; and in pregnant women has often led to abortion." 525.

He does not once allude to the important investigations of the late Dr. Watt, of Glasgow, whose mind was led to the subject by severe domestic losses in his own family. In Dr. Good's methodus medendi, we do not see any thing new, and we think, he leans too much to the antispasmodic, and too little to the antiphlogistic plan of treatment. Bleeding, he thinks, should only be resorted to in severe cases, "as spasmodic affections are often rather increased than diminished by the use of the lancet." It will generally be found better, he observes, to employ blisters as a substitute. The most effectual remedy, he thinks, is emetics, "whose action tends equally to interrupt the return of the paroxysms, and to keep the lungs unloaded, by producing a determination to the surface." The practice recommended by several writers is, also, shortly stated by our author.

Dr. Good has invented the term LARYNGYSMUS STRIDULUS for what is usually denominated spasmodic croup, and which, he observes, differs essentially from real croup. It is thus defined:—"sense of spasmodic suffocation in the larynx, commencing suddenly, and relaxing, or intermitting; cough troublesome; scanty discharge of viscid mucus." In this disease, he says, we have neither inflammation nor the membrane-like secretion of real croup, while the sense of suffocation is produced, not by obstruction, but by spasm.

> "The suddenness with which this complaint commences its attack, forms another mark of distinction between itself and croup, almost as pathognomic as the absence of inflammation, and the peculiar secretion in the latter. There are instances, indeed, in which genuine croup has also commenced abruptly, but these are very rare; for it has usually the precursive symptoms of a slight cough and hoarseness for a day, and sometimes two days, as though the patient were labouring under a catarrh. In croup also, the inflammation, when it has once taken effect, becomes a permanent cause of excitement, and the anxiety and struggle for breath continue with little if any abatement till the inflammation is subdued. In the disease before us, the spasm suddenly subsides in a short time, though it may perhaps return in an hour, or half an hour, or even a few minutes; and in the interval the patient enjoys perfect ease, though the voice is rendered hoarse from the previous straining. Croup is, moreover, an exclusive disease of children; stridulous spasms of the larynx is sometimes found in adults. Those who have been dissatisfied with the name of spasmodic asthma, have, however, treated of it under the name of spasmodic croup, but merely because they have not known how else to distinguish it: for almost every one who has thus noticed it, has acknowledged that it is a different disease, and demands a different plan of cure." 534.

The treatment which Dr. Good recommends is, we believe, judicious. An antimonial emetic should be given as soon as possible, and the diaphoresis which it excites should be maintained for some hours, by keeping the child in bed, and the use of diluents. "The bowels should also be excited by a purgative of calomel." If the emetic does not prove sufficient, or the constriction should be renewed, "laudanum should be exhibited according to the age of the patient, and a blister be applied to the throat." The great danger consists in mistaking this disease for real or inflammatory croup, in which case, although the emetic and purgative, in the first instance, would do no harm, yet the same can not be said of the laudanum.

The genera iii. and iv. dyspnœa and asthma, have afforded Dr. Good ample scope for his favourite discussions, respecting nosological distinctions, etymological definitions, and classical arrangement—discussions that will appear dry and uninteresting to most, and absolutely useless to many of his readers. We sincerely wish he had occupied many hundreds of his pages with recent pathological facts, rather than with ancient or obsolete doctrines.

While discussing some modern hypotheses, our author too often passes over in silence, those that are making the greatest noise in the world. Thus, in debating "whether the suffocative tightness of the chest (in asthma) be the result of a spasmodic stricture of the bronchial vessels, spreading thence to the muscles of respiration; or, produced by an infarction of these vessels, from a superabundant effusion from their exhalents," Dr. Good has taken no notice of the controversy maintained for some years past on the Continent, respecting the pathology of asthma—in which one party, with no mean talent, and many stubborn facts, maintain that the disease is almost invariably dependent on disturbed function or altered structure in the heart. We do not, indeed, subscribe to this doctrine in its fullest extent; but still, we are persuaded, that there is no inconsiderable degree of truth in the melancholy doctrine of Rostan and his followers. In this section, Dr. Good has drawn freely on the standard work of our distinguished countryman Dr. Bree, and, in that, has shewn his judgment. He differs from Dr. Bree occasionally, but in a fair and liberal manner.

Genus vi. is on a disease unknown to, or, at least, undescribed by, the ancients. It has received various appellations from modern writers, as syncope anginosa, asthma dolorificum, orthopnœa cardiaca, and angina pectoris. It is termed sternalgia* by Dr. Good, signifying "breast-pang," which is

---

* Sternalgia was applied to this disease many years ago, by M. Baumes, a French writer. We think, that "coralgia" would be a still more proper term—the sense of distress being, almost always referred to the heart itself.

It is not a little amusing, sometimes, to observe the anxiety of our Parisian neighbours to trace all discoveries which have borne the name of English, to themselves. Thus, in the early volumes of the Dict. des Sciences Medicales, the English are ridiculed for their credulity or ignorance in supposing angina pectoris to be an idiopathic disease; but, by the time they had got to the 52d volume, it was no longer possible to shut their eyes against facts—"l'evidence des faits les a enfin obligés à se retracter." What then was to be done about a disease—"dont la description primitive est généralement attribuée aux Anglais?" What, but claim the discovery for themselves! Four years before Heberden published his account, a *compatriote* wrote a letter to the celebrated Larry, describing angina pectoris. Unfortunately, (there is always some piece of ill luck when the French fail) this letter cannot be found in any *Bibliotheque* in Paris, nor has any author given an analysis of its contents. *Sacre!* It is the only thing wanting to complete the history of French medical literature! "Cette dissertation qu'on doit considerer comme une piece essentielle à l'histoire de la medecine Française." Where is the blame to be thrown for this unaccountable ingratitude to, or negligence of, the poor Peter Perdu? Not on his own countrymen, of course;—no, no—on the *English* certainly! It is entirely the fault of the English that this dissertation is not to be found in the bibliotheques of Paris!—"Pièce que nous devons mettre autant de zèle à faire connaitre, *que les medecins Anglais on ont mis à la laisser dans l'oubli.*" There cannot, of course, be a doubt, that Dr. Heberden travelled all through France, and bought up every copy of this Peter Perdu, which he burnt before he published his discovery. What a happy people are our French neighbours, who can thus draw fuel for their own vanity out of the successes of their rivals.

After all their early scepticism, however, they have at last given a very good account of the disease.

significant enough, as a mere appellation; but, as the disease is now pretty universally acknowledged to be an affection of the *heart*, it certainly appears curious enough, to see it arranged with affections of the lungs. Dr. Good may say, that the function of respiration is disturbed in angina pectoris, but surely so is it, also, in many other diseases, for instance, in pneumonia, which is excluded from this class. Indeed, we are compelled to say, that the farther we proceed, the more convinced we become of the utter hopelessness of any thing like a natural or perfect nosological arrangement of diseases.

Although we are of opinion that the train of phenomena presented in angina pectoris is produced by various and often dissimilar affections of the heart; yet that, in nine cases out of ten, it does depend on *organic* affections of that viscus, we are well convinced. We have seen in our own practice, and through the kindness of professional friends, a good many instances of this disease, and in all (except one case) there was lesions of structure of some kind, in the central organ of the circulation. In the majority of cases there were ossifications or indurations about the valves or vessels of the organ—in some there was an overloaded state of fat—and in others there was a flaccid and easily lacerable condition of the muscular structure of the organ. We have said there was one exception, though we are by no means certain that this was a *real* exception. It was the late Mr. Price, surgeon, of Walthamstow, by whom we were twice or thrice consulted. He had well-marked angina pectoris, and died in one of the paroxysms. We were not present at the dissection, but it was reported that no organic lesion was found.*

On the Continent, indeed, much confusion reigns in respect to angina pectoris. In France they would not acknowledge, till very lately, any such idiopathic disease, and they said that the angina pectoris of the English writers was nothing but spasmodic asthma.† In Italy, Professor Brera of Padua, has written a treatise on this disease, which he designates by the term "*Steno-Cardia*," indicating the oppressed state and impeded functions of the heart produced by the unnatural compression or encroachment of some neighbouring organ. He does not consider the heart itself as the seat of the disease. In most of the cases related by Valerian Brera, there was great enlargement of the liver, causing much encroachment on the region of the heart, and doubtless embarrassing the free action of that organ; but few, if any, of the cases exhibited

* We have since learnt, however, that Mr. Travers found some organic disease.—*Ed.*

† See the article "angine bronchiale," in the Dict. des Sciences Medicales.

the phenomena of real angina pectoris, as known and described in this country. We shall instance the first case as a specimen, somewhat abbreviated.

"A man, 62 years of age, of small stature, but strong and active, had been for several years subject to frequent oppressions suddenly attacking him, which he referred to the middle of the sternum, without feeling any pain in respiration. On the 1st of October, 1804, the oppression returned more frequently and severely than usual, and the patient began to complain of feeling, from time to time, a pricking sensation in the left breast, followed by acute pain, which extended to the left arm. He did not lose his recollection during the attack, which was short, but he became vertiginous, and his sight was confused. When the paroxysm ceased, there remained a sense of numbness in the whole of the left arm; the pulse was more or less irregular, hard and frequent; bleeding was prescribed, but not submitted to.

"On the 22d in the evening, having walked three miles and being fatigued, he was seized with a violent fit of coughing, and expectorated a little bloody mucus.

"On the 28th, he was seized with a violent pain in the head, and fell down senseless; attempts were made to recover him, but he had breathed his last.

"The brain was found in a healthy state; great adhesion had taken place between the right lobe of the lungs and the pleura costalis, as well as between the pericardium and the left lobe. The heart was of its natural shape and texture, except there being some varices along the course of the coronary veins, *and a great dilatation of the right auricle and ventricle.* The liver considerably enlarged and very hard, was displaced from its natural situation, and occupied the whole of the epigastrium; its left lobe was raised in such manner, as to have forcibly pushed up the posterior inferior surface of the heart, and retained that viscus in a state of total compression. The other viscera of the abdomen were in a sound state."

The above case does not correspond with what we have seen in this country under the name of angina pectoris, and after all, as there was actual organic disease of the heart present, we do not see how Professor Brera can decide that the seat of the complaint was in the liver, although it is very probable that the hepatic enlargement conduced to the cardiac affection.

We shall here state the particulars of one of those cases which fell under our own notice, and which we examined after death in the presence of Drs. Lara, Denmark, and some other medical gentlemen.

*Case.* 19*th July.* The reporter was suddenly called to Mr. Myrtle, aged 40, who was found gasping for breath, while his wife was chafing his chest, and another woman his left arm, which appeared pale and lifeless. His face was pallid, but expressive of great distress—lips livid—forehead bathed in cold sweats—pulse rather quick and weak, but not irregular—inability to articulate from pain in the region of the heart and in the left arm, and also from a sense of suffocation. Without waiting for a history of the case a vein was opened, and by the time that 24 ounces of blood were abstracted, the paroxysm ceased. The patient now gave the following history of his case. In April of the year before, while walking in the street, he was suddenly seized with a violent pain in the guard of the left arm, which presently shot into the breast, accompanied by a loss of breath and a sense of dying. He with difficulty got into a house, and swallowed a glass of spirits, soon after which the fit went off. From that time he never passed a day without one or more of these paroxysms more or less severe. They generally come on about five o'clock in the afternoon, while he is walking home from his employment; but they are readily excited at any time by corporeal exertion or mental agitation. For some time past the pain in the region of the heart is accompanied by a distressing palpitation, and a sensation as if the organ was rolling over and over. He says that he sometimes finds the heart cease to beat altogether. The fit generally lasts but ten or fifteen minutes, sometimes an hour or more. There is always, even between the paroxysms, some degree of dyspnœa. The last was the severest he ever had. For some time past he felt, during the fit, a painful kind of globus ascending to the throat; and at all times he is much troubled with flatulence. He is generally costive, and always worse when purged—a paroxysm is not unusual after a lax motion. For two days after the bleeding he had no attack—an unusual immunity; but they then returned as before, and on the night of the 27th of the same month, he was awakened from his sleep by a severe paroxysm, which terminated his existence, about fifteen months from the first accession of the disease.

The body was opened in the presence of Drs. Lara and Denmark. The lungs were healthy—the pericardium contained about an ounce of serum—the heart was large, fat, curiously mottled on its surface, and remarkably soft and flabby. The parietes of the right auricle were peculiarly thin—the auriculo-ventricular opening on that side rather large—no imperfection in the tricuspid valve—right ventricle passively enlarged, its parietes thin and weak—pulmonary artery and valves natural. The left ventricle, which was full of black fluid blood, presented nothing unnatural except the paleness, softness, and weakness of its muscular structure. Incipient points of ossification were observable on the mitral valve. The origin and arch of the aorta were enlarged, and the coats thickened, indurated, and exhibiting scales of ossific deposition, and still broader ones of substance resembling cartilage. The valves were not diseased. The right coronary artery was distinctly seen opening into the aorta, and on being slit open was found natural. The origin of the left coronary artery was sought for, but in vain. A transverse section of the heart was then made, when the left coronary artery was easily recognized, and a probe was passed along it towards the aorta, where its origin was found completely impervious. The whole calibre of the vessel was much smaller than the other, but not otherwise diseased. No disease in any of the abdominal viscera.

Whether, in the above case, the obliteration of the origin of the left coronary artery can be looked upon as leading to the phenomena of the disease or to the softened condition of the muscular structure of the heart, must be a matter of conjecture. That side of the heart could only have been nourished by inosculation with the other coronary artery. It is probable, however, that the obliteration of the origin of the vessel was of recent occurrence, otherwise the shrinking of the rest of the artery would have been still greater than was found to be the case.

Our friend Dr. Palmer, of Tamworth, has stated, about eight years ago, a case which, though it does not appear to have been one of well-marked angina pectoris, is yet very interesting. The following is only an outline.

"Mr. H——, of Staffordshire, nearly 70 years of age, a very stout and athletic man, had been complaining for six or seven months of uneasiness in the left region of the thorax, extending to the loins, right shoulder, and down the arm as far as the elbow. He could not ascend stairs or steep ground without being suddenly arrested with loss of breath and a severe sense of stricture across the lower part of the sternum. He was much troubled with flatulency and cardialgia. In all other respects he seemed the emblem of health and strength, although "his whole life was passed in an alternate state of excitement from immoderate drinking, and of languor and drowsiness from the exhaustion consequent on it." He swallowed, on an average, two bottles of gin daily! His pulse was, in general, weak and frequent; but hurried and irregular on any mental or corporeal excitement. His father and brother had both died suddenly from unknown causes. On the 26th October, 1814, he was well as usual, took dinner with his accustomed appetite, and walked out into an adjoining field, where he assisted to drag a horse out of a well. He complained immediately afterwards of much exhaustion —leaned against a tree—and sunk lifeless on the ground.

Dr. Palmer and Mr. Arrowsmith examined the abdomen and thorax only. The lungs were sound—loose part of the pericardium loaded with fat, but the rest of it healthy—three or four ounces of fluid in the pericardium—heart pale, flabby, softened in structure, and completely empty of blood—pulmonary auricle and ventricle enlarged passively—valves of the pulmonary artery rigid—aortic auricle much dilated—mitral valve thickly strewn along its base with points of ossific deposition—the valve itself had lost its natural structure, and resembled cartilage—aortic valves converted into bone—both coronary arteries were become cylinders of bone—the root of the aorta one-third larger than natural—no ossification of the cartilages of the ribs. The abdominal viscera were sound, and permission could not be obtained to open the head."

We agree with Dr. Palmer that the above case "in its leading characters and termination more strongly resembled angina pectoris than any other organic disease of the heart with which we are acquainted—not, however, that affection in its simple and more rare form, but complicated with valvular disease of the aortic portion of the heart."*

We have made little or no allusion to several writers who regard this disease as totally independent of any organic affection, but rather as occasioned by spasm or the irritation of some translated disease, as gout, &c.†

Among these are Darwin, Berger, Butler, Heberden, Bergius, and Dr. Parr. But dissections are now more frequently and more carefully made than in former periods—and organic diseases are more readily detected. Dr. Good appears to view angina pectoris as "not originating necessarily in any structural derangement of the organs affected," and his nosological definition seems to place it in connexion with the respiratory rather than the circulating organs. In this we cannot agree with our esteemed author. We shall quote his methodus medendi.

"The mode of treatment which I have found most successful consists in putting the patient immediately in an inclined, rather than a recumbent position, with his head raised high. He should instantly take an emetic of whatever may be given most expeditiously, though the antimonial preparations form the best medicine for this purpose, as producing a longer action. As soon as the patient rejects, he may be allowed a little warm water, though this should be administered to him sparingly. The diaphoresis hereby usually induced should be assisted by a moderate warmth of bed-clothes, and particularly by placing the patient between the blankets; and if the constrictive pain or difficulty of respiration still outlast the sickness, opium intermixed with ether, camphor, or other diffusible antispasmodics, should be employed pretty freely." 589.

Our own view of the nature and cause of the disease precludes, of course, all expectation of cure. We have seen the disease retarded in its progress, but no case prevented ultimately from terminating fatally. There are, we acknow-

---

* See the cases of angina pectoris published by Dr. Black of Newry, in the Memoirs of the Medical Society, vol. iv. and also in the 7th volume of the Medico-Chirurgical Transactions, for instances analogous to the above. The experience of Dr. Jenner and Allan Burns also is in favour of the organic cause of the disease. Jurine and Desportes, who have written two excellent dissertations on angina pectoris, consider the disease as attributable to functional disorder of the heart and lungs, and as rangeable among the *neuroses*.

† See New Med. and Phys. Journal for December 1814.

ledge, some cases on record, that seem to prove the curability of the disease. Thus, Dr. Parry himself, who placed its cause in ossification of the coronary arteries (which surely he did not believe to be removeable) mentions the case of an elderly patient who had laboured under angina pectoris, but who had wholly or nearly recovered from the disease by the use of the Bath waters. Credat Judæus! Dr. Robert Cappe also relates a case in the 4th volume of the Medical and Physical Journal, which he asserts was angina pectoris, and cured by nitrate of silver. But we are confident the disease was not angina pectoris. Dr. Cappe never saw the patient in a paroxysm, though the fits always returned once, and sometimes very often daily. We think therefore that Dr. Cappe had very little curiosity or anxiety about the disease at all. In fact, we do not recognize a single symptom of angina pectoris in the case, except that of "gasping for breath," which every body knows attends many other diseases, and winds up almost all. The reasonings which led Dr. Cappe to employ lunar caustic in the cure, we do not well comprehend; but the fact on which they were based was this—that in the year 1793, when galvanising the nerves and muscles of frogs, he found the *latter* restored to energy by touching them with argentri nitras, after they had ceased to obey the electric excitation.

A well-marked case of angina pectoris is related by Dr. Palmer, in the 8th volume of the New Medical and Physical Journal, for December 1814, where considerable alleviation of the disease was obtained by instituting a permanent drain from the region of the heart; by abstinence; quietude of mind and body; moderate exercise in the open air; antacids; bitters; aromatics; and gentle laxatives. These measures attended to during the intervals of the paroxysms, may do much to procrastinate the fatal catastrophe in some cases, though they will prove utterly inefficacious in others.

It may be asked what ought we to do in the paroxysm? Considering the weakened or disorganized state of the heart, we would *not* recommend bleeding, though it will be observed that we bled the patient whose case is narrated. But he appeared to be dying, and we bled him without having time to reflect on the nature of the disease. As it happened, he was relieved by it at the time; but we repeat it that we have since seen bad effects from general bleeding. If called to a patient labouring under the immediate attack, we should be more inclined to exhibit gentle cordials, with cool and fresh air, than any other class of remedial agents. At the same time there may be cases where the heart becomes, as it were, overpowered with blood, as in exercise, &c. and in such cases it may be proper to draw off a few ounces just to relieve the local plethora about the heart for the time. It is curious that great flatulence in the stomach is an almost invariable attendant on angina pectoris, and not a little aggravates the complaint. Indeed we have reason to believe that it occasionally excites the attack. It is therefore of great consequence to obviate this symptom by every mean in our power. Dr. Percival informs us that nothing relieved the paroxysms so instantaneously as venesection and *vomiting;* but death may take place before emetics may be made to act. Dr. Parry, as may naturally be expected, was a strong advocate for sanguineous depletion during the paroxysm, but he desires it to be done under the following precautions:—"blood should be taken from a small orifice, the patient being placed in the horizontal position, while the physician is to keep his finger on the pulse to decide on the limits to which venesection is carried." Rubefacients, frictions, and pediluvia may be serviceable.

CLASS III. *Hæmatica; or Diseases of the Sanguineous Function.* This class contains four orders; viz. fevers, inflammations, eruptive fevers, and cachexies. The physiological proem prefixed to this class descants, 1st, on the machinery of the sanguineous system; 2dly, on the moving powers; and 3dly, on the nature of the fluid circulated. It consists of 33 pages, and exhibits much research, little originality, and some error. We deem the following passage, just under our eye, to be an example of the last ingredient.

"It may be still further observed, that, in a state of inflammation, the pulse of the inflamed part, in consequence of local excitement, is much more frequent than that of the heart or of any other organ. *Thus in a whitlow, the radial artery may give to the finger a hundred pulsations in a minute, while not more than seventy strokes may be exhibited in any other part of the system.* The rapidity of the pulse is in this case usually in proportion to the degree of the inflammatory action; and hence, if the system should labour at the same time under ten different inflammations in different parts or organs of a different structure, as glands, muscles, and membranes, it is possible that it may have so many different seats of pulsation taking place at such different parts at one and the same time, while all of them are at variance with the pulsation of the heart." 17.

We have probably paid as much attention to the phenomena of the pulse and the circulation in general as our brethren, or as Dr. Good himself; but in the course of nearly 30 years' observation, we never witnessed a single instance of the *number* of pulsations in an inflamed part varying from the number of contractions in the left ventricle of the heart.

And if Dr. Good will shew us a single example of the kind marked by us in italics, we will pay him down one hundred guineas—if ragged reviewers can be supposed capable of mustering up such an immense sum in these hard times. We have seen a very few instances, in diseased states of the heart, where the pulsations at the wrist were *less* in number, (generally just half the number) than those at the heart—but never a single instance where they were *more* in number. Indeed we consider the latter to be a physiological impossibility, unless in cases where there is one heart in the chest and another at the elbow. That the diameters of vessels and the *degrees* of their throbbing increase considerably as they approach a phlegmonous tumour or acute inflammation, is well known; but the pulsations are *synchronous* with the other tangible arteries and with the ventricular contractions.

Dr. Good appears to adopt the doctrine that the heart acts in the double capacity of a forcing and sucking pump, and that it is the main, if not the sole moving power of the circulation under ordinary circumstances. He seems also to embrace Dr. Carson's hypothesis respecting the resilient power of the lungs in expanding the chambers of the heart; but as Dr. Carson is very generally allowed to have failed in explaining the circulation of the fœtus in utero, where the said resiliency has not come into play, we think the whole hypothesis must resolve itself shortly into its original constituent principles, like all other things on earth or in the air. It must be confessed that Dr. Good is sufficiently indulgent to new theories and hypotheses—thus Sir Everard Home and others "have established beyond a controversy" the communication between separate organs, "though we can trace no intercourse of vessels." The kindness of the cardiac portion of the stomach in transmitting fluids to the spleen, without the intervention of vessels, is certainly very extraordinary—and still more so the mystic or rather the magic communication between the stomach and bladder. All we can say at present is, that experiments as well as experience are fallacious now as they were in the days of Hippocrates.*

---

* Della Torre made out the corpuscles of the blood to be flat circles or rings with a perforation in the centre—Hewson represents them as hollow or vesicular, with a dot of red colouring matter in the centre, so that they were doubtless "the wheels of life moving on iron axles;"—but "M. Baur," says our author, "has ascertained, he thinks, that it is not the centre of the globule that is dotted, but its outline that is surrounded with colouring matter; so that instead of being annular wheels with iron axles, they are spherular wheels with iron tiers" 33. Risum teneatis amici?

We gladly emerged from the labyrinth of microscopical observations on the blood; but Pyrectica or Fevers stared us in the face, and the very sight of the *word* proved the *thing* contagious, without any arguments, for we instantaneously felt the cold chills of its intermittent form creep over our frame! It requires, indeed, no small degree of courage as well as curiosity to wade once more through the pyrectic theories of the schools;—the concoction and critical evacuations of Hippocrates and Galen—the acid, alkaline, effervescent despumations of Van Helmont—the lentor and error loci of Boerhaave—the spasm of Stahl, Hoffman, and Cullen—the sthenia and asthenia of Brown—the sympathetic associability of Darwin—the inflammatory hypothesis of Marcus—the cephalitis of Clutterbuck—the excitement and collapse of Armstrong—the gastro-enteritis of Broussais—and the new old doctrine of Stimulus and Controstimulus now flourishing in Italy! Yet with all these theories before us, the hue and cry both at home and abroad is—"give us general principles to act upon; and away with your empirical treatment of each case according to its symptoms." If we ask what principle will ye have gentlemen?—one calls out for Armstrong, another for Broussais, a third for Clutterbuck, a fourth for old Cullen, while several sober-looking gentlemen shake their heads, and observe that neither principles nor practice can yet be positively defined, and till then we must "obviate occasional symptoms" as they arise, even at the risk of being designated empirics. In truth, none are greater empirics than those who attempt to act on fixed principles in medicine, where no fixed principles are established. Thus if a Broussaian comes to a fever patient with evident symptoms of determination to the head, and yet, true to his masters's principle, applies leeches to the epigastrium—he is an empiric. If a disciple of Clutterbuck, on the other hand, finds a patient in fever, with pain, tenderness, and fulness about the epigastrium, for which he shaves and blisters the head, *on principle*—he is an empiric. The red hot phlebotomist who bleeds in all cases of fever—because it is fever, is just as much an empiric as his predecessor, the Brunonian, who "never wet a lancet in fever," because all fevers were asthenic. In short, if there be any thing like a well-founded principle in medical science, it is this ridiculed principle of treating every individual case according to the phenomena which it presents at each varying stage, and in each varying constitution, instead of on a pre-established principle which is more likely to be founded in error than truth, and which, if ever so just, could not apply even to the majority of cases.

In the age we live in, two main doctrines respecting fever divide the medical world, one of which is subdivided into certain sects or parties. The first of these doctrines, and that which has still the greater number of adherents, contemplates fever as an idiopathic disease, and though frequently not *necessarily* connected with inflammation of any particular structure in the body. The other doctrine, the disciples of which are increasing, views fever as nothing more than a general or constitutional expression of a local disease—which disease is, of course, inflammation. The converts to this doctrine or reformation have, as is not unusual, split into sects, each asserting that its own dogma is the only infallible one. These sects are three in number. One, the followers of Marcus, regards the local inflammation as sometimes in one organ or structure of the body, sometimes in another—but always present as the basis of fever somewhere.* The second class, with Broussais at their head, have fixed the origin or focus of fever in the mucous membrane of the digestive organs, whence it radiates on all parts of the system, and produces the phenomena hitherto falsely attributed to a kind of entity or imaginary being denominated idiopathic fever. The third party of phlogotics (as they may be called in contradistinction to the pyrectics, or idiopathic party) are pretty numerous in this country, and consider Dr. Clutterbuck or Dr. Ploucquet as their founder. It is well known that they confine the *fons et origo* of fever within the parietes of the cranium and vertebral canal, making phlogosis of the brain, its membranes, or its appendages the exclusive proximate cause of fever.

Now it is quite impossible that these various doctrines can be all right. And yet we think they are all right—*at times.* It is the Procrustic bed, on which each party stretches or cramps his favourite theory, that deprives them all of their natural proportions, and renders them all more or less deformed.

Of the various theories of fever, we believe that those which give most latitude to the causes, most variety to the features, and most contingency to the consequences or post-obit phe-

* "On this ground it is that Professor Marcus of Bavaria, who has contended with similar strenuousness for the identity of fever and inflammation, has regarded *all inflamed organs as equal causes;* and is hereby enabled to account for the different kinds of fever that are perpetually springing up before us. Thus inflammation of the brain, according to Marcus, is the proximate cause of typhus; inflammation of the lungs, of hectic fever; that of the peritoneum, of puerperal fever; and that of the mucous membrane of the trachea, of catarrhal fever." *Good*, vol. ii. p. 59.

nomena, will come nearest the truth. We consider, therefore, that the *first* doctrine to which we have alluded (though not without faults, some of which we shall presently point out) approximates most to a true estimate of the nature of fever. We do not think that the fundamental principle of this doctrine has ever been more clearly or more succinctly stated than in the following words of Dr. Fordyce :—

" 'Fever, therefore,' says he, 'is a disease that affects the whole system; it affects the head, the trunk of the body, and the extremities; it affects the circulation, the absorption, and the nervous system; it affects the skin, the muscular fibres, and the membranes; it affects the body, and affects likewise the mind. *It is, therefore, a disease of the whole system, in every kind of sense.* It does not, however, affect the various parts of the system uniformly and equally; but, on the contrary, sometimes one part is much affected in proportion to the affection of another part.' "*

The validity of the above doctrine has not, we imagine, been shaken by all or any of the investigations which have taken place since the days of Fordyce. The statement quoted will certainly apply to fever when regularly formed; but if we look more narrowly into the chain of phenomena which connects this state with the first application of the cause of the fever, we shall probably be inclined to relax a little in favour of those doctrines which assign a "*local* habitation and a name" to the disease. If a person, for instance, be carefully watched after exposure to the febrific miasm issuing from marsh or human sources, he will unquestionably exhibit phenomena that indicate a local or circumscribed sphere of morbid action, in the earliest movements of the disease. In whatever way the poison gets into the system, the sensorium commune, as might naturally be expected, appears first to feel its effects. Our own observations and our own feelings would induce us to think that the *mental* faculties or functions are impaired at an earlier stage than any of the grosser functions—implying that the brain and nervous system are primarily implicated. It is very easy to see that any functional ailment in this class of organs (whose influence on all parts of the body is so extensive) must so very promptly derange the various other functions of the human frame as to render priority of lesion quite undistinguishable by the great mass of observers—and thus give rise to the idea of general and simultaneous disorder throughout the whole system. Thus far then we agree with Ploucquet and Clutterbuck in making the brain and its appendages the primary seat of

* On Fever, Dissert. i. p. 28.

fever. But so far from considering this primary affection as inflammation, we regard it as quite the reverse—at least the phenomena presented by it (the only proofs we can have in that stage) are diametrically opposite.

When, indeed, that operation takes place in the economy which has aptly enough been termed *reaction*, a state obtains in the brain, the viscera, and the vascular system, very closely allied to, and very often terminating in, unequivocal phlogosis. Whether we designate this condition (which precedes, resembles, and, we apprehend, induces inflammation) by the terms excitement or irritation, is not perhaps very material. It is known in the living body by a lower degree of those functional disturbances which attend idiopathic inflammation —and in the dead body by a turgescent state of the capillaries of the part. Both this state and the actual inflammation into which it often runs, are also known to the observant practitioner by a therapeutical phenomenon—namely, that, whereas you may *control* them by the usual antiphlogistic means, you will very seldom be able to *remove* them with the same facility and in the same space of time, that you would idiopathic inflammation—and why? because they are produced by an effort of the constitution to resist or repel a deleterious agent—which effort requires, in general, a cycle or period varying according to the nature of the morbific agent and the powers of the constitution. The poisons of plague, of typhus, of variola, and other diseases offer ample proofs and illustrations of this position.

That local inflammations do frequently occur in fevers of every description, and in almost all protracted and fatal cases, is now proved beyond the possibility of doubt, by the great disturbance of function in the organ or organs before death, and the manifest alterations of structure on dissection. Death, however, does occasionally take place without leaving any trace of inflammation—and that in three different ways, viz. 1st, where the force of the febrific cause or miasm is so great, or the constitution so weak, that no regular reaction of the system ensues, and life is destroyed by a gorged state of the vessels of the brain and viscera. 2dly. Where the reaction is so violent that the great vital functions are exhausted or overwhelmed before any of the usual appearances of inflammation have had time to form. 3dly. Where the disease is long protracted, from whatever cause, and the patient gradually sinks by a slow exhaustion that leaves little or no trace of structural alteration after death.

If then these local inflammations be contingencies or consequences rather than causes or essential concomitants of the fever, it may be asked why do they take place in one organ or class of organs rather than in others—or why do they take place at all? It may be answered, that when the cause of fever (say the poison of typhus, variola, or plague) is applied to the constitution in sufficient quantity, that process termed reaction of the whole, but especially of the vascular system, must take place, consistent with the laws and safety of the animal economy. But this reaction or great vascular commotion is not without its attendant danger. Even in the best constitutions, where every organ seems endued with its just proportion of power, and the equilibrium of the functions is perfect, the whole system experiences a great shock during fever, and the patient is left in a state of vast debility and exhaustion. What then is naturally to be expected when one organ or class of organs is previously weak or imperfect in its functions? We see the consequence too often, when the vascular excitement of measles, variola, or scarlatina—nay, the common symptomatic fever from a wound or accident, excites phthisis in persons whose lungs are previously unsound. Upon this principle only can we account for the balance of irritation or inflammation inclining sometimes to one organ, sometimes to another in fever—and we see it almost invariably fall upon that structure in greatest force, which was previously predisposed to disorder from the habits or original constitution of the patient. The alimentary canal and the brain are unquestionably the organs most frequently affected in fever, as we might naturally expect, from the intemperate habits and turbulent passions of society. The followers of Ploucquet and Clutterbuck will more easily persuade themselves than others that the brain is the primary organ affected, and the other lesions merely secondary. On the other hand, the Broussaians may argue for the priority in the abdominal viscera; while the unbiassed observer will probably join us in the view which we have taken of the contingency of inflammation in fever. Indeed, we think that a careful consideration of the various remote *causes* of fever;—of the various *forms* which it assumes in different epidemics, in different climates, and in different subjects—of the various *modes of treatment* which have succeded in the disease—and of the various *post-obit appearances* recorded by practitioners, might have deterred any but the most determined theorists from setting up such circumscribed systems of pathology as Drs. Ploucquet, Clutterbuck, and Broussais have done.

The foregoing brief exposé of the nature of fever may not be inappropriately followed by a few words on the general principles of treatment. A considerable range of observation, and a very attentive consideration of the subject have long since led us to compress the voluminous *principles* laid down by authors into a very narrow compass—we had almost said into a nutshell. Viewing fever then as an effort of Nature to resist some morbid impression or expel some morbid miasm, we consider it the grand business of the practitioner to watch the several *functions*, and by relieving them when oppressed, thus protect the *structure* of the parts. In this office he will sometimes be obliged to control the action of excited organs, and excite the action of torpid ones—and if there be a general principle in the treatment of fever, this is the one most universally applicable at the bed-side of the patient. This principle scarcely requires elucidation to render it comprehensible by the meanest capacity; but we shall apply it to a few of the leading phenomena of fever.

We come to a patient at a very early period of fever, and where the individual, for instance, has been exposed to the contagion of typhus or concentrated marsh poison. We find the surface cold and pale, the pulse weak and quick, the breathing hurried and laborious, a sense of oppression at the precordia, disturbed intellect, diminished secretions. What can be more evident than that the whole balance of the circulation and of the excitement is here deranged. The skin and secretory organs are torpid—and the blood is oppressing the vital viscera. What does the principle in question point to under such circumstances?—To the very best mode of treatment. Apply the tepid bath to the surface to draw the circulation there—give warm diluting drinks for the same purpose—and act upon the various abdominal secreting organs, and on the skin, in order to restore their functions, which are for the time suspended.

But in a short time we see the whole phenomena reversed, as it were. We find the surface burning, the face flushed, the arteries throbbing, the capillaries injected, the intellect excited to delirium, and still the secretory organs locked up. Here then we are obliged to almost exactly reverse the means we were before pursuing. The surface must be cooled by fresh air or gelid water—blood must be drawn if the excitement run high, or any one organ appears to suffer more than the others—and the bowels must still be acted on. By these means, and without these means by the powers of Nature, a third state or condition of the system occurs, differing materially from either of the preceding. The skin and the secreting organs act, and the whole phenomena of fever suffer a great diminution or entire, though temporary, solution. In this third state what are the indications?—To do nothing at all. But in the fourth state or remission, what do the *principles* of our systematic sticklers point to? To the use of an empirical remedy, bark or arsenic, which their principles never discovered or dreamt of, and cannot even now explain!

But instead of these stages or conditions of the system being pretty distinctly marked, and sufficiently regular in their succession, we have too ofteu, as in continued fevers, a medley of all the stages at the same moment, one part of the system being in a state of collapse or torpor, another in a state of excitement, and a third in a state of relaxation. Now in these amalgamations we do fearlessly aver that we have no other safe rule to follow but that of counteracting symptoms as they arise, and as they happen to predominate in one organ or set of organs more than in others. Our paramount duty, we repeat it, is to diligently watch the *functions* of the various organs, and, whenever any of them appear to labour disproportionately, to endeavour to relieve them by drawing off the current of the circulation and of the excitement by local or by general means. By a careful conduct of this kind, and by avoiding the MEDECINA PERTURBATRIX, except upon very urgent occasions, we shall give Nature the fairest chance of going safely through the peculiar cycle which the disease, the constitution, or the climate may require for the work of restoration.

The long laboured, and almost endless descriptions of the ever varying forms, features, and shades of fever, (which are never the same in two individuals) are far better calculated to make a book than a practitioner. In the first place they cannot be retained in the memory; and in the second place, they would be useless if they were retained. The simple rules which we have sketched out here are those which we have found most applicable at the bed-side of sickness, and we apprehend that they will prove of no mean advantage to the young practitioner. We have, of course, only alluded to the indications, leaving the means of fulfilling these indications to the choice or discretion of the medical attendent. They are sufficiently well known.

And now to return to Dr. Good. In his observations on the remote causes of fever, he seems to coincide with Cullen that the two great sources are human and marsh effluvium, viz. contagion and a certain aerio-terrestrial influence or agent to which we have given the name of marsh effluvium or malaria. Dr. Good very properly remarks that there are a very considerable number of other causes than the above, which are capable not only of predisposing to, but of exciting the disease, as cold, intemperance, heat, the passions, and numerous other agents. We cannot agree entirely with the following sentiment of our author.

"And notwithstanding that it becomes us to speak with diffidence upon a subject respecting which we are so much in want of information, I may venture to anticipate that the evidence to be advanced in the ensuing pages upon the general nature and diversities of fever, will show that there is more reason for believing that the febrile principle produced by marsh and human effluvia is a common miasm, only varying in its effects by accidental modifications, and equally productive of contagion, than that it consists of two distinct poisons, giving rise to two distinct fevers, the one essentially contagious, and the other essentially uncontagious, as contended for by Dr. Cullen." 64.

Although we are advocates for *contingent* contagion in certain cases of marsh fever; yet, considering the very marked difference which almost invariably obtains between these and fevers from human contagion, we cannot admit the identity of miasm in both classes of disease. We agree with Dr. Good that "the marsh and oozy soil of inhabited countries is necessarily a combination of animal and vegetable matter," a fact to which most of our writers on marsh fevers seem, of late, to have shut their eyes, for they speak of nothing under such circumstances, but decayed or decaying vegetable substances; yet the product of this vegeto-animal poison we look upon, if the expression of its effects is to be taken into account, as different from the poison generated by the living body labouring under disease, as, for instance, under typhus fever.

With the exception of the 4th corollary in the following summary, we agree with our learned author in the justness of his conclusions.

"1. The decomposition of dead organized matter, under the influence of certain agents, produces a miasm that proves a common cause of fever.

"2. The whole of these agents have not yet been explored; but so far as we are acquainted with them, they seem to be the common auxiliaries of putrefaction, as warmth, moisture, air, and rest, or stagnation.

"3. The nature of the fever depends, partly upon the state of the body at the time of attack; but chiefly upon some modification in the powers or qualities of the febrile miasm, by the varying proportions of these agents in relation to each other, in different places and seasons. And hence, the diversities of quotidians, tertians, and quartans; remittent and continued fevers, sometimes mild and sometimes malignant.

"4. The decomposition of the effluvium transmitted from the living human body, produces a miasm *similar to that generated by a decomposition of dead organized matter*, and hence, capable of becoming a cause of fever under the influence of like agents.

"5. The fever thus excited, is varied or modified by many of the same incidents that modify the miasmic principle when issuing from dead organized matter; and hence, a like diversity of type and vehemence.

"6. During the action of the fever thus produced, the effluvium from the living body is loaded with miasm of the same kind, completely elaborated as it passes off, and standing in no need of a decomposition of the effluvium for its formation. Under this form it is commonly known by the name of febrile contagion. In many cases, all the secretions are alike contaminated; and hence, febrile miasm of this kind is often absorbed, in dissection, by an accidental wound in the hand, and excites its specific influence on the body of the anatomist.

"7. The miasm of human effluvium is chiefly distinguishable from that of dead organized matter, by being less volatile, and having a power of more directly exhausting or debilitating the sensorial energy, when once received into the system. Whence the fevers generated in jails or other confined and crowded scenes, contaminate the atmosphere to a less distance than those from marshes and other swamps, but act with a greater degree of depression on the living fibre.

"8. The more stagnant the atmosphere, the more accumulated the miasmic corpuscles from whatever source derived; and the more accumulated these corpuscles, the more general the disease.

"9. The miasmic material becomes dissolved or decomposed in a free influx of atmospheric air; and the purer the air the more readily the dissolution takes place; whence, è contrario, the fouler as well as more stagnant the air, the more readily it spreads its infection.

"10. Under particular circumstances, and where the atmosphere is peculiarly loaded with contamination, the miasm that affects man, is capable also of affecting other animals.

"11. By a long and gradual exposure to the influence of febrile miasm, however produced, the human frame becomes torpid to its action, as it does to the action of other irritants; whence the natives of swampy countries, and prisoners confined in jails with typhus contamination around them, are affected far less readily than strangers; and, in numerous instances, are not affected at all.

"12. For the same reason, those who have once suffered from fever of whatever kind hereby produced, are less liable to be influenced a second time; and, in some instances, seem to obtain a complete emancipation." 77.

We cannot enter on a consideration of the individual species and varieties of fever detailed in an able and interesting manner by our author, in the first 200 pages of the second volume. The student will there find a great deal of information collected from various sources—the lecturer, much to furnish out his discourse to his pupils—and the practical physician something to smile at, occasionally, in the conflicting doctrines and hypotheses brought forward each in its turn, "to strut and fret its hour upon the stage."

The third order of this class embraces the subject of inflammations, and occupies more than 300 pages of letter press. It is not much to be expected that Dr. Good should loose or cut the Gordian knot of inflammation. We are much mistaken if the following summary of "the little that we know upon the subject" might not have been comprised in a still smaller space than our author has allotted to it.

"The little that we know upon the subject may, perhaps, be comprized in a few words:—The standard of firm health is the best guard against inflammations of every kind, or the state in which a man is least susceptive of them; and a deviation in either direction, whether towards a habit of *entony* or of atony, *capacifies* him for breeding them. But it does not *capacify* him equally; for in the latter case they are produced far more easily and generally than in the former. In *fibrous entony*, obstruction appears to take place, and inflammation to follow, from an increased tendency to constriction and rigidity in the muscular tunic of the arteries generally, and an actual constriction in those of the part affected; in consequence of which the diameter of the tube is diminished, and the blood, though urged by a stronger impetus from behind, works onward with less freedom than usual. In fibrous atony, obstruction takes place from the relaxed and yielding state of the vessels which admit grosser corpuscles of the blood than what naturally belong to them, and thus become accessary to the error loci of the Boerhaavian school. But a mere error loci is not sufficient for inflammation: since the erratic corpuscles are readily forced back, or pass diagonally into larger vessels from the numerous anastomoses that prevail in the arterial system. Of this we have a pertinent example in the red suffusion which frequently takes place in the tunica albuginea of the eye, which is often an effect of weakness alone, is unaccompanied with heat or pain, and consequently with inflammation, and perhaps passes off by the next day. In addition, therefore, to the relaxed state of fibres and the error loci before us, there must be something of that irritability which is so frequently an attendant upon relaxed and mobile organs, and which produces spasmodic and contractile action in a far higher degree, though, perhaps, in irregular fluxes and refluxes, than any habitual firmness or rigidity of fibre does at any time." 233.

If Dr. Good was able to form any clear idea of the foregoing theory of inflammation, it is far more than we have been able to do. Well might he say in the succeeding paragraph, "concerning the proximate cause of inflammation there is yet much to be unravelled." We have marked in italics a few words which shew Dr. Good's propensity towards unusual terms and expressions.

Our author has given us a tolerably fair statement of what we know respecting the remote causes, and a few of the laws which seem to govern inflammation; yet even in this portion of the work we think we could "pick some holes in his skirt," were we inclined to be critical. Thus in our author's zeal to shew the "wise and beneficent laws of Providence," as exemplified in the tendency of suppurations to point outwards, we imagine Dr. G. has rather overstepped the mark.

"Thus," says he, "if an inflammation attack the peritoneum covering an intestine, and adhesions are hereby produced between the two, the inflammatory action works upwards through the thick walls of the abdominal muscles, while the proper coats of the intestines in most instances remain sound." 239.

Now in the course of nearly 30 years' experience, we do not recollect to have ever seen an instance where, in peritoneal inflammation, the inflammatory action worked its way outwards; but we have seen many instances where erosions of the intestinal coats obtained, and where there was extravasation of fœcal maters into the common cavity of the abdomen. So far then from Dr. Good's position being the general rule, we should be inclined to look upon it as the exception.

Again, in the next page, our author lays it down, that "simple or *healthy inflammation* is a *remedial process* for restoring a part to soundness, when affected by a morbid impression that has a tendency to injure or destroy it." Yet, in the same page, when speaking of the treatment, he lays it down as a fundamental principle, that we should endeavour "to make a new impression upon the part, and to oppose a healthy or remedial, to an *unhealthy and mischievous, action.*" Here then, *healthy inflammation*, which is a *remedial process* in the beginning of the page, is an *unhealthy and mischievous action* at the close of it. There appears some incongruity in these statements.

Of the individual inflammations it will not be in our power to speak. In general Dr. Good has amassed a great body of information on each particular subject; but here and there we observe a very defective state of his pathological researches. This defect is particularly conspicuous in his chapters on inflammation of the lungs and their investing membranes. Thus, in peripneumony we have the terminations in effusion, suppuration, and gangrene: but he does not notice the consolidation or hepatization, as it has been called, of the pulmonary structure; a very common termination of inflammation, acute and chronic. The following passage contains the whole of our author's pathology of pleurisy.

"Like the preceding species also, pleurisy terminates in resolution, suppuration, and gangrene. The former is the ordinary and most favourable issue. The last occurs rarely; but suppuration is by no means uncommon; in which case, if the abscess do not point outwardly, an empyema will necessarily follow; and the formation of pus is indicated by a remission of the pain, one or more shivering fits, and, in some instances, a sense of fluctuation. This, however, is a termination far more common to pleurisy from external injuries, than from internal causes." 366.

Yet, in nine cases out of ten of fatal pleurisy, the termination is by sero-purulent effusion into the cavity of the chest, without any thing like an abscess pointing outwardly. It is well ascertained, also, that in all cases of acute pleuritis, there is more or less of serous effusion, which becomes absorbed, in the event of recovery, leaving or not, according to the extent of the disease, adhesions between the pleura pulmonalis and pleura costalis. In a new edition, Dr. Good must extend greatly his pathological researches; and on the subject of pulmonic affections, he cannot draw from a better source than the investigations of Laennec and Baron.

By the way we have some fault to find with Dr. Good in his treatment of pulmonary inflammation. "The best, the easiest, and even the natural cure of peripneumony is expectoration." We cannot subscribe to this doctrine. It is the *natural* cure no doubt, because Nature has no other way of unloading the vessels, without great danger. But by a prompt and decisive depletion we will very often save Nature the trouble of even expectoration—at least the inflammation may be frequently subdued before any expectoration appears, which will then be very trifling in quantity.

Again, Dr. Good takes no notice of antimony in the treatment of pneumonia, excepting as an occasional auxiliary to saline draughts in determining to the skin and promoting diaphoresis. "One of the most common, and, at the same time, most useful refrigerants, is nitre; which may be combined with the citrate of potash, or made to produce a more certain determination to the skin by the addition of camphor or of antimonial wine, or by a combination with the citrate or acetate of ammonia." This is all that is said of antimony as a powerful controller of the circulation, taken in doses just sufficient to produce nausea, especially when aided by digitalis, another important remedy which we do not observe in Dr. Good's methodus medendi.

Our author, however, strongly recommends the action of full vomiting, (by what medicine is not said) and that kept up for an hour or two, even when the pulmonic inflammation "has made considerable advance." Not having given the measure a trial, it would not be fair to condemn it; but, we confess that, beyond nausea, we should be afraid to proceed in inflammation of such an organ as the lungs or their investment.

Our author notices the dangerous phenomenon of suppressed expectoration in the progress of pneumonia. We have seen it caused by an incautious cathartic, and we know of no medicine so powerful in restoring it, as the carbonate of ammonia in doses of ten grains frequently repeated.

We shall pass on to the subject of dysentery. It is always disagreeable to us to take up the review of systematic works, because analysis is not applicable to them, and criticism is the paramount duty of the reviewer in such cases. It is his main object, also, to point out what he conceives to be errors or imperfections, for the good both of the author and public; what he passes unnoticed being considered as having his approval. This is the path which we are unavoidably compelled to pursue in the work before us; and if our articles are chiefly composed of censures, it is because we cannot possibly even enumerate the chapters and sections of valuable matter diffused through these volumes.

The dissertation on dysentery not only disappointed, but almost provoked us. During the last memorable war, our fleets and armies were dispersed over every part of the globe, and our medical officers collected a mass of important information on the subject in question, such as had never before been collected or recorded. Yet, not a single writer of the last twenty or thirty years does our author seem to be acquainted with,—or, if acquainted with, has he noticed. Dr. Harty, who some fifteen or twenty years ago, made dysentery the theme of his inaugural dissertation at Edinburgh, is the only author quoted since the antiquated publication of Mosely some thirty years ago! Even Harty is only noticed as having accidentally fallen into our author's hands, after part of his chapter on dysentery was composed. In fact, it appears to us that Dr. Good, not liking to take the trouble of looking around him to see what was the existing state of our knowledge on the subject of dysentery, as scattered through all our periodicals and many distinct and excellent monographs during the last twenty years, has contentedly fallen back upon Sydenham, and determined "to take him as our polar star"*—Sydenham who, it is our firm belief, never examined a dead body after he left his academical studies—at least he has given us no indication of pathological knowledge in any of his works!

We shall not enter into particular criticism on a chapter

* See page 449 of vol. ii.

which, we are most reluctantly constrained to say, is unworthy of our author. In the etiology and pathology there is no information at all—and what little is said of practice is, generally speaking, erroneous, bad, and antiquated. In another edition Dr. Good had better cancel the whole of this chapter, and consult those modern authors who saw and felt dysentery in various climates—who dissected their patients, and consequently became acquainted with the pathology of the disease—who discarded the unfounded Cullenian notion of contagion—who treated the malady with *success*, notwithstanding the dogma of Mosely to the contrary—and who, finally, have exhibited a more enlightened *ratio symptomatum* than his favourite Sydenham did, whose genius could only reach the sublime theory, that dysentery "was a fever turned in upon the guts."

The next subject discussed by Dr. Good is *bucnemia sparganosis*, or phlegmatia dolens. The only authors quoted are White, Trye, Ferriar, Hull, and Denman. He makes no allusion to the able investigation of Dr. Dickson of Clifton, in the second volume of the quarterly series of this journal; nor does he advert to the circumstance that phlegmatia dolens has occurred in the unimpregnated state, and even in the male. An instance of this last was met with in a marine by Dr. Denmark, then surgeon to Haslar Hospital, who has published the case and a plate of the limb—another instance happened in the person of Dr. Purdy of New York—and a third in the practice of the late Mr. Baynton of Bristol. Bating these deficiencies in the history of the disease, Dr. Good's account, though short, is sufficiently correct, and the therapeutic plan judicious. The latest information which we have been able to obtain respecting the pathology of the disease is, that it depends, sometimes at least, on inflammation of the great inguinal vein. A physician-accoucheur of this metropolis has some preparations, we believe, shewing this circumstance, and means to publish on the subject.

Acute rheumatism occupies but seven pages of the volume under review, which is by far too short a space, when we consider the importance of the subject. Dr. Good has taken no notice of that very dreadful disease, rheumatic metastasis to the heart, which, for many years past, has occupied much the attention of the medical world.

In the chapter on gout we see nothing that need detain us. It also is rather short, when we consider what ponderous tomes have been written on the subject. Dr. Good, who appears to have had some visitations of the disease in his own person, seems not disinclined to a limited application of cold to the part arthritically inflamed; but acknowledges that he has never been able to muster up courage to put in execution that *striking* peculiarity of practice recommended, some years ago, by Dr. Balfour, namely, percussion and compression. "But our sheet-anchor," says he, "is opium; and it should be given freely, and in union with some preparation of antimony, so as to act towards the surface generally, and thus restore to the living power its interrupted equilibrium."

On the subject of the exanthematous diseases, including plague, there is a very fair body of information brought forward by our author, but nothing novel, of course; we must therefore pass over this entirely, and here close our article, having already overstepped our boundary. In a future number we shall pursue our examination of these volumes.

